# Comments on “Vitamin Pharmacogenomics: New Insight into Individual Differences in Diseases and Drug Responses”

**DOI:** 10.1016/j.gpb.2017.09.001

**Published:** 2017-10-07

**Authors:** Tom Greenfield

**Affiliations:** International College of Generative Medicine, Canterbury, Kent CT1 1BL, United Kingdom

Dear Editor,

I would like to offer some comments on the excellent article by Hai-Yan He and colleagues published in *Genomics, Proteomics & Bioinformatics* on 1st April 2017 [1]. The authors include, in the list of genetic polymorphisms that have an effect on vitamins, the low concentrations of cellular and plasma vitamin B_12_ in GG carriers of SNP rs602662 (772 G > A) in the gene encoding fucosyltransferase 2 (*FUT2*).

The G allele of *FUT2* rs602662 is the ancestral allele, and as reviewed in the article by He and colleagues, has been recognised in various genome-wide association study (GWAS) analyses as linked with lower plasma levels of vitamin B_12_ [1]. This SNP is in strong linkage disequilibrium with the FUT2 W143X nonsense variant (rs601338) [2], which is known as the primary allele for non-secretor status of ABH blood group antigens [3]. The ancestral (G) allele of SNP rs601338 allows for normal translation of *FUT2* (ABH secretor), whereas the variant A allele, linked with the missense variant rs602662(A), results in a premature stop codon in *FUT2* and a truncated, dysfunctional FUT2 enzyme, leading to the ABH non-secretor phenotype.

The association between *FUT2* and vitamin B_12_ is likely to result from ABH non-secretor status, as the association between plasma vitamin B_12_ concentration and the *FUT2* rs492602 (in perfect linkage disequilibrium with rs601338) is found to be stronger than that for rs602662 [3].

The secretor phenotype was originally believed to be associated with reduced plasma B_12_ due to an association with *Helicobacter pylori*-induced gastritis [4], but this was refuted in a later study by Oussalah and colleagues which investigates the influence of *FUT2* polymorphism on *H. pylori* serologic status [5]. A subsequent paper by Chery et al suggests that FUT2 phenotype worsens B_12_ status by decreasing gastric intrinsic factor (GIF) secretion via a mechanism related to H-type fucosylation, particularly in the presence of *GIF* mutations, and is unrelated with *H. pylori*-induced gastritis [6].

Interestingly, a recent GWAS analysis by Nongmaithem and colleagues based on Indians in the Pune Maternal Nutrition Study has identified allele-specific differences for rs78060698 in *FUT6* [7]. This variant is found to differentially influence the binding affinity of hepatocyte nuclear factor 4α (HNF4α), and the knockdown of *HNF4α* may repress the expression of *FUT3*, *FUT5*, and *FUT6*. This in turn would affect production of the fucose moiety in the glycan structure secreted in the intestinal tract, which has an important role in maintaining host-microbial interaction, as well as microbial abundance and diversity. Thus, the rs78060698 variant could play a part in mediation of intestinal host-microbial interaction, leading to alteration of plasma B_12_ concentrations. Given the influence of the *FUT2* ABH non-secretor variant on plasma B_12_ levels, and also the difference between vegetarians and non-vegetarians [8], this seems entirely feasible. Nongmaithem et al also consider the possibility of an influence of FUTs on B_12_ concentrations, which may be mediated through the glycosylation of B_12_-binding proteins and their receptors including transcobalamin I, GIF, cubilin, and CD320. The authors state however that the mechanism through which the FUTs act upon these B_12_-binding proteins and their receptors is unclear and needs to be investigated further.

## Competing interests

The author has declared that no competing interests exist.
